# The vocal development of the pale spear-nosed bat is dependent on auditory feedback

**DOI:** 10.1098/rstb.2020.0253

**Published:** 2021-10-25

**Authors:** Ella Z. Lattenkamp, Meike Linnenschmidt, Eva Mardus, Sonja C. Vernes, Lutz Wiegrebe, Michael Schutte

**Affiliations:** ^1^ Department Biology II, Ludwig Maximilians University Munich, Martinsried, Germany; ^2^ Graduate School of Systemic Neurosciences, Ludwig Maximilians University Munich, Martinsried, Germany; ^3^ Neurogenetics of Vocal Communication Group, Max Planck Institute for Psycholinguistics, Nijmegen, The Netherlands; ^4^ School of Biology, St Andrews University, St Andrews, UK

**Keywords:** vocal learning, deafening, vocal development, auditory feedback, hearing impairment

## Abstract

Human vocal development and speech learning require acoustic feedback, and humans who are born deaf do not acquire a normal adult speech capacity. Most other mammals display a largely innate vocal repertoire. Like humans, bats are thought to be one of the few taxa capable of vocal learning as they can acquire new vocalizations by modifying vocalizations according to auditory experiences. We investigated the effect of acoustic deafening on the vocal development of the pale spear-nosed bat. Three juvenile pale spear-nosed bats were deafened, and their vocal development was studied in comparison with an age-matched, hearing control group. The results show that during development the deafened bats increased their vocal activity, and their vocalizations were substantially altered, being much shorter, higher in pitch, and more aperiodic than the vocalizations of the control animals. The pale spear-nosed bat relies on auditory feedback for vocal development and, in the absence of auditory input, species-atypical vocalizations are acquired. This work serves as a basis for further research using the pale spear-nosed bat as a mammalian model for vocal learning, and contributes to comparative studies on hearing impairment across species.

This article is part of the theme issue ‘Vocal learning in animals and humans’.

## 1. Introduction

Human vocal development and speech learning require acoustic input and auditory feedback mechanisms, which allow the gradual modification of utterances to ultimately match a previously perceived auditory target [1]. Humans who are born deaf or severely hearing impaired never acquire a normal adult speech capacity [2]. Instead, the vocal development of deaf infants shows variations such as reduced inventories for consonants, vowels and syllables, reduced articulation space, and atypical temporal and coordinative sound production [3,4]. Few other animal taxa show a similar dependence on auditory feedback for their vocal development and those that do are considered capable of vocal production learning. Vocal learning animals (e.g. elephants, pinnipeds, cetaceans, bats, songbirds, parrots and hummingbirds [5]) are able to directionally modify their vocalizations based on auditory input [6]. Bats have been highlighted as a promising mammalian model for the study of vocal learning [7,8]. The pale spear-nosed bat, *Phyllostomus discolor*, possesses a rich vocal repertoire [9], is flexible in the spectro-temporal domain of its vocalizations [10,11], and is able to vocally adjust its calls towards acoustic templates [12,13].

Here, we investigated the impact of the absence of auditory feedback on the vocal development of *P. discolor*. Complementing a previous isolation study with juveniles (i.e. one that disrupted external auditory input), which focused on vocal adjustment towards playbacks of a single vocalization type [14], we investigated the effect of deafening (i.e. severely disrupting acoustic perception and thus auditory feedback) on their vocal development. We acoustically deafened juvenile bats (at 9–11 days of age) and demonstrated that this hearing loss was permanent via auditory brainstem recordings (ABRs). We compared the vocal development of the deafened bats with age-matched hearing control animals, which allowed us to assess the impact of auditory feedback on the vocalizations of this bat species.

## 2. Material and methods

### (a) Animals

The pale spear-nosed bats, *P. discolor*, originated from the breeding colony of the Ludwig Maximilian University Munich. In our experiments, six bats (four females, two males), all born between 26 January and 8 February 2017, were studied. They were housed with a mixed population of 24 juvenile and adult, male and female conspecifics in a colony room from birth. For details on the individual bats, see electronic supplementary material, table S1. The experiments were approved by the German Regierung von Oberbayern (approval no. 55.2-1-54-2532-126-2016).

### (b) Deafening protocol and hearing assessment

We used acoustic overstimulation to severely impair the hearing capacity of three juvenile bats (less than two weeks old). The deafening was performed by presenting the anaesthetized bats with intense (140 dB peak-equivalent sound pressure level) frequency modulated sweeps (1–45 kHz) played back in a continuous loop for 2 h. The acoustic signals (sampling rate 192 kHz) were converted to analogue by an audio interface (Fireface UTC, RME, Haimhausen, Germany) and amplified by an audio amplifier (AVR 347, Harman Kardon, Stamford, CT, USA). For details on the anaesthesia and medication protocol see the supplementary material. To test the hearing capacity of all six bats (i.e. deafened experimental bats (*N* = 3, all female) and hearing controls (*N* = 3, two males, one female) (figure S1)), ABRs were performed following Linnenschmidt & Wiegrebe [15]. For details on the ABR recording and analysis, see the electronic supplementary material.

### (c) Acoustic data acquisition

The acoustic recording of the six bats was performed at two life stages: as juveniles (within the first six months of their lives) and as adults (at about 2 years of age) (electronic supplementary material, table S1). Data acquisition from the juvenile bats was conducted in pairs, consisting of a focal juvenile and its respective mother, being 1 m apart from each other, separated in pyramidal nets (electronic supplementary material, figure S2A). Vocalizations of each pair were recorded continuously for 20 min sessions initially three times per week (months 1–3), and then once per week (months 4–6), cumulating in 53 recording sessions per pair. The acoustic data of the mothers were not analysed in this study.

The acoustic recordings of vocalizations of the six bats at adult age (electronic supplementary material, table S1) were conducted in two groups of three individuals (separated into deafened and control group) in a different acoustic set-up, i.e. a small, instrumented box (electronic supplementary material, figure S2B), which was installed in an acoustic chamber. For details on the recording set-up and acoustic data acquisition see the supplementary material. Each group (experimental and control) was recorded in five sessions of 2 h length, leading to 10 h of recordings per group.

### (d) Acoustic data analysis

Vocalizations were automatically detected and extracted from the recordings with the help of a custom-written MATLAB script. In order to be classified as a vocalization, a vocal emission had to be at least 20 dB louder than the background noise. Additionally, the vocalization had to be separated from a previous or subsequent vocalization by at least 20 ms (juveniles) or 5 ms (adults) of silence, adjusted for optimal data extraction. In the analysis, we exclusively focused on social vocalizations by excluding all vocalizations with a duration of less than 3 ms (e.g. echolocation calls) [16]. Thirteen spectro-temporal parameters of every vocalization were extracted or calculated [9]. Here, we focus on the five most commonly used parameters: vocal activity, amplitude, duration, fundamental frequency and aperiodicity. A statistical summary of all extracted parameters for the juvenile (9–11 days and 2–25 weeks of age) and adult recordings is given in electronic supplementary material, tables S2, S3 and S4, respectively. As we used two different acoustic recording set-ups for juvenile (i.e. mother–juvenile pairs with restricted mobility) and adult (i.e. freely moving, group) recordings, we did not compare these values in one statistical comparison. Moreover, the identity of the vocalizing animal could only be determined in the juvenile recordings, allowing exclusively descriptive statistics for the adult data (electronic supplementary material, table S4). Based on the juvenile data (2–25 weeks of age; after deafening of the experimental bats), we tested each parameter for significant differences between the three hearing and three deafened individuals, for significant changes due to age, and for significant interactions of these two parameters (mixed-model ANOVA with treatment group and age as fixed factors and subject identity as a random factor; *p*-values controlled for multiple testing using the Benjamini–Hochberg procedure [17]; table 1 and electronic supplementary material, table S3).

**Table 1.** Five spectro-temporal parameters of juvenile bat vocalizations. The vocal parameters were extracted from deafened and hearing juvenile *Phyllostomus discolor* vocalizations at 2–25 weeks of age, i.e. after deafening of the experimental bats. For a list of all analysed parameters see electronic supplementary material, table S3. *N*: number of vocalizations. Q25–Q75: interquartile range. Q50: median, s.d.: standard deviation. *f*_0_: fundamental frequency. D/H: deafened versus hearing. I: interaction. *p-value < 0.05. Significant differences between the three hearing and three deafened individuals (D/H), change due to age (age), and significant interactions (I) of these two parameters were assessed with a mixed-model ANOVA with treatment group and age as fixed factors and subject identity as a random factor. *p*-values are controlled for multiple testing using the Benjamini–Hochberg procedure [17].

vocalization parameter	deafened (*N* = 317 941)				hearing (*N* = 24 227)				*p-*value		
	Q25	Q50	Q75	mean ± s.d.	Q25	Q50	Q75	mean ± s.d.	D/H	age	I
vocal activity (calls per 10 s)	5.2	13.6	28.4	17.0 ± 14.2	0.0	0.3	1.7	1.7 ± 3.4	*	0.05	0.05
amplitude (dB)	13	20	28	21 ± 10	25	31	36	29 ± 9	*	*	*
duration (ms)	3.4	4.3	7.2	9.4 ± 12.6	14.6	35.4	52.3	33.9 ± 21.0	*	0.89	0.89
mean *f*_0_ (kHz)	17.7	19.9	21.0	19.3 ± 3.7	13.6	15.0	16.3	14.9 ± 3.1	*	*	*
aperiodicity (1)	0.10	0.28	0.39	0.25 ± 0.17	0.01	0.02	0.08	0.06 ± 0.09	0.06	*	*

## 3. Results

We assessed the hearing capacity of the six bats, in the form of audiograms via ABRs, over the course of the first six months of life and at 3 years of age (electronic supplementary material, figure S1). We confirmed that hearing was severely impaired in the experimental group after deafening and was not recovered within the period tested. Hearing developed and was retained normally for the control group. Both deafened and hearing juveniles physically developed normally (based on external observations), were nursed by their mothers, learned to fly and were apparently socially integrated in the colony (i.e. they were roosting in social groups, fed normally, and showed no overt signs of harassment; M Linnenschmidt, EZ Lattenkamp 2017–2018, personal observations). However, we did not specifically investigate their social behaviour in groups and social stressors could have varied between the groups.

Five vocal parameters of the pale spear-nosed bat (vocal activity, amplitude, duration, fundamental frequency and aperiodicity) were compared between the deafened experimental group (*N* = 3) and the hearing control group (*N* = 3). All individuals were born within two weeks of each other, and were recorded as juveniles (over the first 25 weeks of life) and as adults (approx. 2 years of age). The vocal parameters analysed here were extracted from the acoustic recordings of these six bats at both developmental stages. From a total of 106 h of recordings (i.e. 354 726 vocalizations) from the juvenile bats, 91.1% of vocalizations (323 180) were emitted by deafened bats and only 8.9% (31 546) by hearing bats (electronic supplementary material, tables S2 and S3). In 20 h of recordings (20 152 vocalizations) from adult bats, 98.6% (19 874) were emitted by the deafened and 1.4% (278) by the hearing bats (electronic supplementary material, table S4).

**Figure 1.**
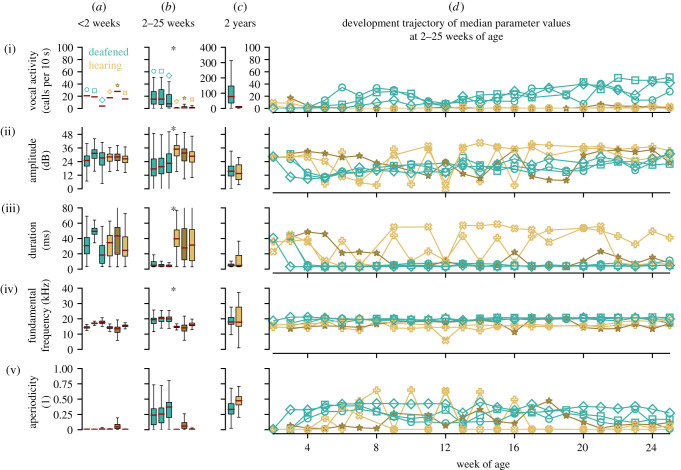
Vocal parameters of the deafened (*N* = 3) and hearing (*N* = 3) bats. Deafened individuals (three females) are presented in green, while the hearing bats are presented in brown (two male individuals are indicated in light brown and one female is indicated in dark brown). (*a*–*c*) Depicted are boxplots representing the extracted vocal parameters at different ages. (*a*) Data acquired during a single recording session at less than two weeks of age, before the experimental animals were deafened. (*b*) Data acquired at 2–25 weeks of age, after the experimental animals were deafened. Asterisks (*) indicate significant differences between the hearing and deafened groups as assessed using a mixed-model ANOVA. (*c*) Data acquired from the same bats at adult age in five recording sessions per group. (*d*) The individual developmental trajectory of the median values of the vocal parameters at 2–25 weeks of age (i.e. same data as in *b*).

Vocal activity (number of calls per 10 s) was similar for all bats very early in life (less than two weeks, figure 1*a*(i)), but increased strongly in the deafened juveniles during development (figure 1*b,d*(i) and table 1). This increased vocal activity was persistent in adulthood, with the deafened bats producing 98 calls per 10 s on average (figure 1*c*(i)), compared with less than 20 calls per 10 s emitted by the hearing control group (figure 1*c*(i)). Initially, all bats used similar vocal amplitudes (figure 1*a*(ii)). During development, vocalization amplitude of the deafened juveniles was generally lower than that of the hearing bats (figure 1*b*(ii)), but increased steadily over the recording period (figure 1*d*(ii) and table 1). In adulthood, the vocal amplitudes were similar again (figure 1*c*(ii)). Call duration was similarly varied for both groups early in life (figure 1*a*(iii)). However, call duration decreased substantially in the experimental group (figure 1*b,d*(iii)). Call duration of the juvenile bats was not dependent on age or interaction, but instead varied strongly between the two treatment groups (table 1). The fundamental frequency, or pitch, of the emitted vocalizations was similar for both groups (15–20 kHz) and remained relatively constant throughout their development (figure 1*a–d*(iv)). The tendency of the experimental bats to emit higher-pitched calls (figure 1*b,d*(iv)) did not persist into adulthood (figure 1*c*(iv)). The aperiodicity or spectral roughness of the calls was generally low for all bats in the first weeks of recording, meaning that most of the early vocalizations produced were relatively tonal (figure 1*a*(v)). As development proceeded, the deafened juveniles generally emitted calls with higher aperiodicity (figure 1*b,d*(v)), but not so as adults (figure 1*c*(v)). The vocalizations emitted by the deafened individuals were more similar to each other than to the vocalizations of the controls (figure 1*b,d* and table 1).

## 4. Discussion

In the current study, three juvenile bats were deafened at less than two weeks of age, and their vocal development was studied in comparison with a control group, consisting of three age-matched, normally hearing conspecifics. We found that the deafened group showed increased vocal activity and their vocalizations were substantially altered, being much shorter, higher in pitch, and more aperiodic during development than the vocalizations of the hearing control animals, suggesting that hearing is important for the normal vocal development of these bats.

If deafened animals display normal vocal development this demonstrates that auditory input is not necessary to shape their vocal repertoire and rules out vocal production learning [18,19]. On the other hand, while the deafened pale spear-nosed bats did display modified vocal development, this does not conclusively prove the occurrence of vocal learning in this species, until other effects of deafening, such as reduced social interactions and stress, can be ruled out. Furthermore, it is important to recognize that some types of change to call production in the absence of auditory input is not exclusive to vocal learning species. For example, reduced vocal activity has been observed for deafened and isolated squirrel monkeys [18,20], but increased vocal activity has been observed in both deaf human children [4] and deafened infant guinea pigs [21]. Similarly, beyond four weeks of age, deafened bats showed a dramatic increase in vocal activity compared with the control group, which persisted into adulthood (figure 1*b–d*(i)). It is conceivable that the increased vocal activity is generated in an endeavour to compel conspecifics to socially interact with them, although this has not been directly tested. Call amplitude is another parameter that can be affected in deafened animals of vocal learning and non-learning species. The deafened juvenile bats steadily increased the amplitude of their vocalizations over the course of their development and were ultimately marginally louder than the adult control group (figure 1*c,d*(ii)). It is likely that increased call amplitude is an effect of overcompensation for the lack of auditory feedback and may not be related to the learning of species-typical vocalizations. Vocal changes in deafened animals can thus also occur in vocal non-learning species owing to a number of other external influences (e.g. social learning, group integration and stress levels).

**Figure 2.**
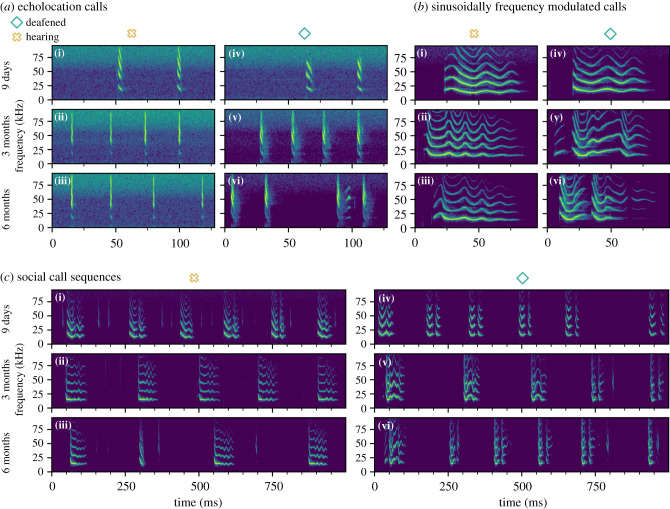
Example spectrograms illustrating vocal development of one hearing (i–iii) and one deafened (iv–vi) bat over the first six months of their lives. Three types of vocalizations are depicted: (*a*) echolocation calls, (*b*) sinusoidally frequency modulated calls, and (*c*) social call sequences. Each vocalization type is shown at three different developmental stages of the bats: at 9 days (i and iv), at three months (ii and v) and at six months (iii and vi) of age.

A number of consequences of deafening in pale spear-nosed bats observed herein were consistent with those characteristic of other vocal learning species. In addition to the increased vocal activity noted above, hearing impaired children display differences in phonation, reduced use of canonical syllables and increased duration of utterances, hypothesized to be due to reduced control resulting from a lack of babbling. Songbird vocalizations are also strongly affected by removing auditory input, since severely reduced vocal repertoires are observed in deafened zebra finches [22,23], sparrows [24] and canaries [25]. We found that the duration of vocalizations emitted by deafened bats was much shorter than those of hearing conspecifics, and control animals showed much greater variation in the length of their calls (figure 1*b*(iii) and table 1). While this difference is not as prominent in adult bats, the variability in call length is still higher in hearing bats (figure 1*c*(iii), and electronic supplementary material, table S4). As call fragmentation is a common occurrence in the deafened bats and a likely cause for their emission of short calls (figure 2), we think it is probable that, in this species, vocal learning may be involved in the acquisition of more complex, long-duration social calls. This is in contrast to findings in vocal non-learning species. Deafened chickens developed a full repertoire of vocalizations and showed no difference in frequency or temporal characteristics of calls or variability of calls compared with their hearing counterparts [26]. Similarly, genetically deafened mice show no differences in the usage or structure of vocalizations compared with their hearing littermates [27,28]. Deafened pale spear-nosed bats can still produce calls with some species-specific characteristics (figure 2); however, the calls are abnormally short, and the calls are more aperiodic, demonstrating the importance of auditory input for the development of normal vocalizations in this species. Overall, the vocalizations emitted by the deafened individuals are more similar to each other (figure 1*b,d* and table 1), consistent with an observation of Romand & Ehret [29] that deafened cat vocalizations are generally more uniform. This is likely caused by the lack of conspecific acoustic input and the resulting limited vocal repertoire. It is generally assumed that auditory feedback is also required for fine tuning of vocal emissions later in life, as has been demonstrated, for example, in bats [30], humans [31], and adult Bengalese [32] and zebra finches [33]. However, experimental evidence of vocal degradation due to deafening in adulthood is still lacking for *P. discolor*.

It is important to note that all deafened bats in this study were female, while the control group consisted of mixed sex animals (i.e. one female and two males). Although *P. discolor* is known to have a rich vocal repertoire [9], to date nothing is known about vocal sexual dimorphism in this species. While *P. discolor* females use maternal directive calls [14], this call type has also been recorded from males of this species [11]. Females of the closely related species *Phyllostomus hastatus* were reported to use screech calls to identify group members [34], but the use of these syllables in males has not been investigated to date and the sex-specificity of these calls is still debated. However, in another phyllostomid bat species, *Carollia perspicillata*, certain trill types have only been recorded from male individuals [35]. Similarly, in other bat species, e.g. from the Emballonuridae family (such as *Saccopteryx bilineata*), distinct vocal sex-dimorphism has been reported in adults, but not in juveniles [36]. Considering the limited behavioural context in the recordings (i.e. mother–juvenile pairs), sex-specific vocal behaviours are unlikely to have an effect on the vocal development data. This assumption is supported by the strong similarity shown between the call parameters of all three control animals, despite there being one female control animal (figure 1, indicated in dark brown), and two males (figure 1, indicated in light brown). Despite the current lack of evidence for sexually dimorphic vocal behaviour in this species, the adult recordings could be influenced by the difference in behavioural context (i.e. the experimental group comprised females only, while the control group was mixed) and possible sex-specific vocal behaviours. As these recordings were conducted in groups of three, assignment of individual calls to a specific animal was not possible. Investigating the occurrence and possible effects of sex-specific differences in the vocal behaviour of *P. discolor* presents an interesting future research avenue.

In summary, we show that deafened *P. discolor* acquire and in parts maintain species-atypical vocalizations, demonstrating that pale spear-nosed bats rely on auditory feedback for normal vocal development. This work further contributes to comparative studies on the effects of hearing impairment across species and highlights the usefulness of bats for the study of mammalian vocal learning.

## Data accessibility

All data and scripts used in this study can be found online and are accessible via the online repository G-Node: https://doi.org/10.12751/g-node.lrqd92 [37].
